# Initiated Breastfeeding and Physiological Patterns in Newborn Infants When Reunited With Mother After Separation Due to Elective Cesarean Birth

**DOI:** 10.1111/birt.12880

**Published:** 2024-10-06

**Authors:** Ana Ayala, Kerstin Erlandsson, Kyllike Christensson, Eva Christensson, Gabriel Cavada, Marianne Velandia

**Affiliations:** ^1^ Department of Women's and Children's Health, Division Reproductive Health Karolinska Institutet Stockholm Sweden; ^2^ Ministry of Health Santiago de Chile Chile; ^3^ School of Health and Welfare Dalarna University Falun Sweden; ^4^ Departments of Physiology and Pharmacology Karolinska Institutet Stockholm Sweden; ^5^ School of Public Health, Faculty of Medicine University of Chile Santiago Chile; ^6^ School of Health, Care and Social Welfare Mälardalen University Västerås Sweden

**Keywords:** caesarean section, full‐term infants, skin‐to‐skin care

## Abstract

**Background:**

The purpose of this study was to compare the effects of two caregiving models on full‐term healthy infants' wakefulness, rooting and sucking reflexes, initiation of breastfeeding, and physiological parameters when reunited with their mothers after a mother–infant separation of 130 min after elective cesarean birth.

**Methods:**

Ninety‐five mother–infant pairs participated in a randomized controlled trial, in which full‐term healthy infants were allocated to be either dressed in their mothers' arms (*n* = 56) or skin‐to‐skin with their mother (*n* = 39) when reunited with the mother within 130 min after cesarean birth. Data were collected by the Neonatal Behavioral Assessment Scale (NBAS) to assess the infants' wakefulness and prebreastfeeding behaviors. Physiological parameters were assessed at 15 min intervals, from 130 to 205 min after birth. Time to first breastfeed was measured in minutes from the reunion with the mother.

**Results:**

The primary finding was that physiologic parameters did not differ but time for initiation of breastfeeding after the reunion with the mother was significantly faster in the skin‐to‐skin group compared to the infants in the mothers' arms group (*p* = 0.005). Over the full study period, a more relaxed state and drowsy were found in the skin‐to‐skin group compared to the infants in the mothers' arms group.

**Conclusion:**

Healthy full‐term infants born by elective cesarean, who were cared for by their mothers when reunited within 130 min of separation and cared for by their fathers during the mother–infant separation, initiated breastfeeding successfully and showed stable physiological patterns.

## Graphical abstract

Over the observation period, skin‐to‐skin infants displayed a more relaxed and drowsy state compared to those held dressed in their mothers' arms. There were no significant differences in vital signs between the groups. Infants in the skin‐to‐skin group initiated breastfeeding significantly faster (*p* = 0.005) than those in the mothers' arms group.

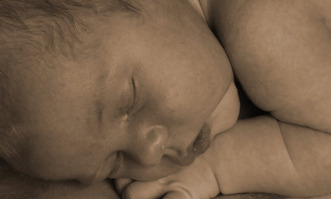

## 1 Introduction

Improving the quality of care around the time of birth, especially in low‐ and middle‐income countries (LMICs), has been identified as the most impactful strategy for reducing stillbirths, and maternal and newborn deaths [1]. In the immediate postpartum period, the quality of care could be improved if all newborns without complications, including low‐birth‐weight babies, were placed skin‐to‐skin with their mothers during the first hour after birth to prevent hypothermia [2]. Previous studies have found that infant temperature becomes stable when the infant peacefully goes through the nine phases of behavior during skin‐to‐skin contact with their mother immediately after birth; these include birth crying, relaxation, awakening, activity, crawling, rest, familiarization, sucking, and sleep [2, 3]. Early physical contact reduces crying and promotes breastfeeding [3]. Furthermore, it increases parental sensitivity to the infant's behaviors [4, 5]. Skin‐to‐skin contact between mother and newborn supports bonding, reduces time to first breastfeed, maintains the temperature of the newborn, and reduces stress and formula supplementation in hospitals [6], promoting optimal development [7].

The child's health and well‐being could be promoted by being offered the breast as soon as possible after birth when the mother and the baby are both ready and clinically stable [8]. The baby‐friendly hospital initiative with zero separation empowers and enables parents to take on their primary responsibility for the child [9] and thus ensure the rights of the child [10]. Exclusive breastfeeding is higher after 1 to 15 min of skin‐to‐skin contact between mother and newborn [11]. Our point of departure is that in many hospitals around the world, mother–infant separation is still common [12, 13, 14, 15], particularly after an elective cesarean birth. When they are clinically stable, the reunion of the mother and child might be supported by a midwife who provides the child early skin‐to‐skin contact with the mother and with assistance to initiate breastfeeding. This is in line with the WHO recommendations [8]. As an alternative, the mother will be reunited with a dressed baby in her arms, but in such a position that initiation of breastfeeding might be delayed [16]. In previous publications derived from our research program in a Chilean hospital, the father's presence was important to both the mother and the father [17, 18], and a stable physiological pattern for the newborn infants was found, with some advantages for the skin‐to‐skin cared for infants [19]. The advantage of skin‐to‐skin care with the father versus being cared for in a cot and the effects on the infant have been shown in a randomized control trial from 2007. The infants in the skin‐to‐skin group were comforted; they stopped crying, became calmer, and reached a drowsy state earlier than the infants in the cot group. It was concluded that the infants conserved their energy in these important hours after birth and when reunited with the mothers the infants were in an alert state ready to initiate breastfeeding [20]. To our knowledge, different care models at the reunion with the mother, skin‐to‐skin versus dressed in the mother's arms, have not previously been studied. Thus, the aim of this study was to compare the effects of two caregiving models on full‐term healthy infants' wakefulness, rooting and sucking reflexes, initiation of breastfeeding, and physiological parameters when reunited with their mothers after a mother–infant separation of 130 min after elective cesarean birth.

## 2 Material and Methods

### 2.1 Design

A randomized control design with randomization in two steps was used.

### 2.2 Study Setting

**FIGURE 1:**
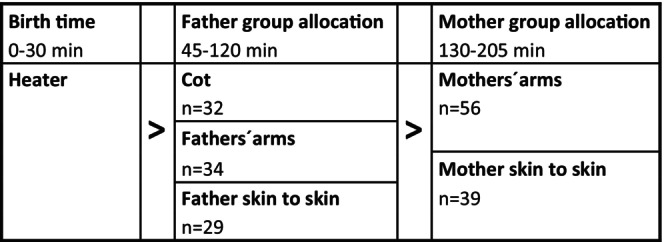
Flowchart of study design allocation.

The study was conducted in Chile from 2009 to 2012 at a public maternity hospital with 9000 deliveries per year. At the time of the study, fathers were not allowed to be present in the operation theater or the postsurgery unit. After the cesarean birth, mothers were transferred to the postsurgery unit for observation, and the infants were transferred to the neonatal unit to be placed under a heater for 30 min [19]. See Figure 1.

### 2.3 Inclusion Criteria

Included in this study were healthy mothers with an uncomplicated pregnancy. The cesarean births were performed with epidural anesthesia due to previous cesarean or by birthing person's request for elective cesarean. The newborn infants were included if they had a gestation age of between 37 and 42 weeks and an Apgar score of > 7 at 1 and 5 min after birth, with a temperature of at least 36.5°C, and assessed as healthy at the first check‐up 30 min after birth. The inclusion criteria for fathers were that they had expressed a wish to take care of the infant while they were separated from their mother after the cesarean birth.

In order to detect a difference between the groups, having a Type I error of 0.05 and a power of 0.80, a professional statistician calculated that at least 11 participants were required for each group: the difference in mean value 5.0 and the standard deviation 4.0.

### 2.4 Randomization

The day before the elective cesarean, 130 prospective parents were approached in consecutive order and informed about the study, out of which 107 couples agreed to participate. Twelve infants did not meet the health inclusion criteria 30 min after birth and were excluded from the study. The randomization of the 95 (73%) remaining infants was performed using sealed, opaque envelopes that contained information about group allocation: (1) cot, (2) fathers' arms, or (3) skin‐to‐skin with the father [19]. After being cared for by the father, a second randomization of the 95 infants (100%) was performed into two groups for the reunion with the mother, either (1) skin‐to‐skin or (2) dressed in the mother's arms.

The first randomization of 95 infants was performed within 45 min after birth after the health checkup of the infant 30 min after birth. The second randomization of the same 95 infants was performed 120 min after birth. The newborn was transferred to the mother at the obstetric unit and placed either skin‐to‐skin (*n* = 39) or dressed in mother's arms (*n* = 56) within 130 min after birth. See Figure 1.

### 2.5 Procedures

Immediately after the cesarean birth, the umbilical cord was cut, and the infant was dried off. If assessed as necessary, oral suctioning of mucus from the infant's mouth was conducted in both groups. The Apgar score was measured from 1 to 10, at 1 and 5 min after birth. The infant was, according to routine procedures, wrapped in two towels and shown to the mother, then cared for under a heater for 30 min (see Figure 1), and then examined, measured, and weighed by a midwife. The ear temperature was noted. The infant was wearing a cotton diaper and covered with two blankets during this assessment and then transferred to the neonatal unit with the father. The mother, in turn, was transferred to the postsurgery unit. The fathers were free to spontaneously interact and care for their child without limitations, except for the fathers in the cot group, who were asked not to pick up the infant during mother–infant separation. In the 45 to 120 min after birth when the infant was with the father, the infant was observed for a 75 min period with no additional nutrition provided and no blood sugar measured. The observations consisted of infant's physiological variables (ear temperature, heart rate, and peripheral oxygen saturation), and their state of wakefulness was evaluated during the first minute of every 15 min interval [19]. After a second randomization into two different mother groups 120 min after birth, the newborn infant and the father went to the obstetric unit from the neonatal unit, to be reunited with the mother. During the mother group allocation 130 to 205 min after birth, the infant was observed for a 75‐min period (Figure 1).

The infants allocated to the *skin‐to‐skin group* (*n* = 39) were reunited with the mother, dressed in a cotton diaper, and placed in skin‐to‐skin contact with their mother at her chest. The infants allocated to the *mother's arms group* (*n* = 56) were dressed in diapers, cotton pajamas, and cotton caps, and then placed in the mothers' arms.

The mothers in both the skin‐to‐skin group (*n* = 39) and the mother's arms group (*n* = 56) were lying down in the bed supported by a pillow, and both the mother and infant were covered with a blanket. Mothers in the skin‐to‐skin group and mothers in the mother's arms group were free to spontaneously interact and care for their children without limitations and were encouraged to start breastfeeding. The mother's spontaneous interaction with her child, except for time of first breastfeeding, were not documented by the observer.

After the observation period, at 205 min after birth, the mother and the infant in both groups—the skin‐to‐skin group and the mother's arms group—were transferred to the maternity ward for conventional care [19].

### 2.6 Data Collection

During the mother group allocation, the infants' wakefulness, rooting and sucking reflexes, time for initiation of breastfeeding, physiological parameters (ear temperature, heart rate, and peripheral oxygen saturation), and their state of wakefulness were evaluated during the first minute of every 15 min interval [19] for 75 min from 130 to 205 min after birth. The Neonatal Behavioral Assessment [21] (NBAS) (Table 1) was used to measure the infants' state of wakefulness, rooting, and sucking, with six observations, at 15 min intervals, for each infant. The first author, or a trained research assistant (here called observer), conducted the naturalistic observations and performed all the measurements to ensure the reliability of the study [19].

**TABLE 1:** The NBAS scoring scheme for infant state of wakefulness, rooting, and sucking [21].

Variable	State	Code	
Wakefulness	Sleep	Code 1	Deep sleep, regular breathing, eyes closed, no activities
	Sleep	Code 2	Light sleep Eyes closed, rapid eye movements, low activity level
	Drowsy	Code 3	Eyes may be open, dull, heavy‐lidded, closed, delayed responses to stimuli, not alert
		Code 4	Bright look, focused, attention to stimulation, motor activities minimized, glazed look
		Code 5	Eyes open, motor activities, fuzzy vocalizations
	Crying	Code 6	Crying that is difficult to break through with stimulation with typical “crying” face, cupped tongue
Rooting		Code 0	Absence of lip or tongue movements
		Code 1	Weak lip movements
		Code 2	Half turns to grab with open mouth
		Code 3	High rooting and mouth movements
Sucking		Code 0	Absence of movements
		Code 1	Barely suction
		Code 2	Medium rhythmic suction
		Code 3	Exaggerated suction

The infants' ear temperature was measured using the Braun ThermoScan thermometer (Braun GmbH, Kronberg, Germany). Heart rate and peripheral oxygen saturation were measured on the infants' index fingers with a Saturator Masimo, which is a compact pulse oximetry device (Masimo Corp, California, USA). Time to first breastfeeding was measured in minutes from the reunion with the mother. Demographic data and data on the health of the mothers and infants were obtained from the birth records and registered in the research protocol [19].

### 2.7 Data Analysis

Data were inputted from chart to a computer before analysis was performed. All variables were normally distributed, and the analysis was performed using a predetermined protocol. The continuous variables at nominal level and the physiologic parameters at ordinal level were described by means and standard deviations to compare differences between the groups and for each time and measurement. We evaluated trends in the responses and the comparison between the two caregiving groups through overtime analysis of variance for repeated measurements. Statistical significance was set at 0.05 and calculated with t‐tests [19]. All data were processed in Stata version 14.0 (Stata Corp LLC, Texas, USA). This study was approved by the Ethics Committee of the Scientific Assessment. Metropolitan Health Service South East (Dnr 16‐05‐2008).

## 3 Results

### 3.1 Demographic Characteristics

For demographic characteristics and baseline data of the infant, see Table 2. The mothers of the infants were healthy multiparas with an average of two previous children between 0 and 5 years of age. They were of Chilean origin and on average 29 years old (SD ± 6.3). There were no significant differences between the ages of the mothers in the skin‐to‐skin group (*n* = 39) and the mother's arms group (*n* = 56).

**TABLE 2:** Demographic and baseline data on the infants.

The infants'	Skin‐to‐skin group (*n* = 39)	Infants in the mother's arms group (*n* = 56)	*p*
Gestational age (weeks)	39.0 (SD ± 0.99)	38.9 (SD ± 0.90)	0.819
Girls	19 (49%)	32 (57%)	0.368
Boys	20 (51%)	24 (43%)	0.368
Weight girls (g)	3476 (SD ± 413.52)	3492 (SD ± 428.36)	0.816
Weight boys (g)	3630 (SD ± 276.40)	3488 (SD ± 454.26)	0.410
Apgar score	9	9	ns
Infants' temperature	36.8°C	36.8°C	ns
Time to first breastfeed (minutes)	2.66 (SD ± 2.88)	6.33 (SD ± 8.38)	0.005*

* *p* ≤ 0.05 is considered statistically significant.

### 3.2 The Behavior of the Infants

#### 3.2.1 Prefeeding Behavior and Time to First Breastfeeding

The infants' prefeeding behavior was assessed with the NBAS (Table 1), and time of first breastfeeding over the study period was assessed for the infants in the skin‐to‐skin group (*n* = 39) and in the mother's arms group (*n* = 56). Over the full period (130 to 205 min after birth), sucking movements were significantly lower (*p* = 0.033) in the skin‐to‐skin group (Mean 0.74, SD = 0.99) compared with the mother's arms group (Mean 1.01, SD = 1.14). Time to first breastfeeding in minutes after the reunion with the mother was significantly faster in the skin‐to‐skin group (Mean 2.66, SD = 2.88) compared to the infants in the mother's arms group (Mean 6.33, SD = 8.38, *p* = 0.005) (Table 2).

#### 3.2.2 Wakefulness

**FIGURE 2:**
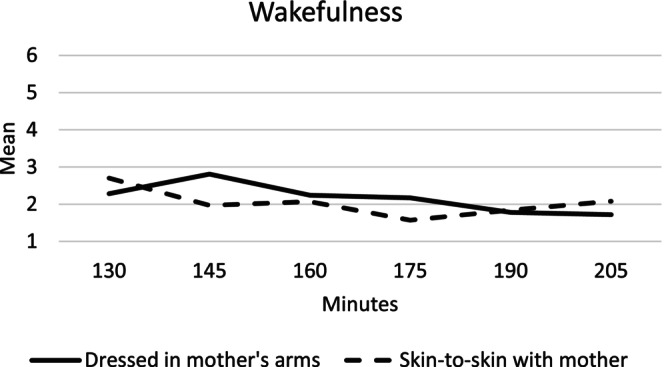
Mean wakefulness for the infants 130–205 min after birth in the mother's arms and skin‐to‐skin groups.

The infants in the mother's arms group showed a significantly higher state of wakefulness at 145 and 175 min after birth compared to the infants in the skin‐to‐skin group (Figure 2 and Table 3). Over the full study period, a more relaxed state was found in the skin‐to‐skin group (Mean 2.04, SD = 1.32) compared to the infants in the mother's arms group (Mean 2.28, SD = 1.52, *p* = 0.063). We also observed the pattern of wakefulness within each group as a function of time. Infants in the skin‐to‐skin group maintained a sleep state from 145 min and throughout the observation period. The pattern also changed in the mother's arms group; from 190 min, the infants maintained a sleep state. The infants' state of wakefulness is presented in Figure 2 and Table 3.

**TABLE 3:** Wakefulness, rooting, and sucking movements for infants in the mothers' arms and skin‐to‐skin groups.

NBAS	Mothers' arms group	Skin‐to‐skin group	*p*
	(*n* = 56)	(*n* = 39)	Skin‐to‐skin vs. mothers' arms
	Mean (SD)	Mean (SD)	
Wakefulness			
130 min	2.28 (1.13)	2.70 (1.30)	0.052
145 min	2.81 (1.61)	1.97 (1.09)	0.006*
160 min	2.24 (1.36)	2.07 (1.41)	0.413
175 min	2.17 (1.53)	1.57 (0.93)	0.032*
190 min	1.78 (1.16)	1.84 (1.50)	0.595
205 min	1.72 (1.22)	2.08 (1.49)	0.069
Rooting			
130 min	1.77 (1.18)	1.375 (1.2)	0.127
145 min	1.43 (1.27)	1.025 (1.18)	0.119
160 min	0.98 (1.27)	1.025 (1.16)	0.865
175 min	1.13 (1.23)	0.45 (0.90)	0.004*
190 min	0.56 (0.96)	0.74 (1.11)	0.428
205 min	0.60 (0.99)	0.83 (1.16)	0.338
Sucking			
130 min	1.60 (0.92)	1.15 (0.78)	0.006*
145 min	1.32 (1.13)	0.80 (0.99)	0.023*
160 min	0.92 (1.08)	0.82 (0.91)	0.562
175 min	1.03 (1.20)	0.45 (0.78)	0.009*
190 min	0.52 (0.87)	0.61 (0.90)	0.770
205 min	0.59 (0.73)	0.59 (0.81)	0.980

* *p* ≤ 0.05 is considered statistically significant.

#### 3.2.3 Rooting

**FIGURE 3:**
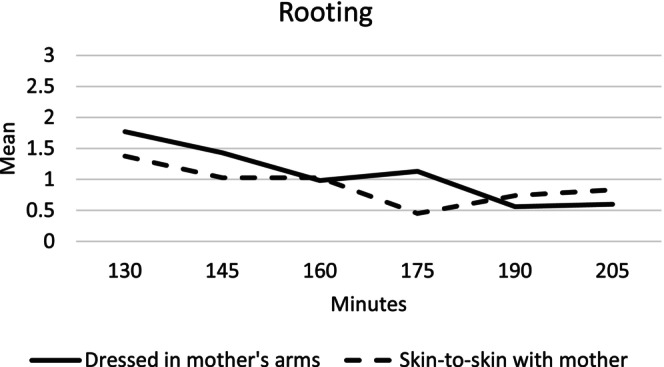
Mean rooting movements at 130–205 min after birth for infants in the mother's arms and skin‐to‐skin groups.

The mother's arms group showed a significantly higher state of rooting at 175 min compared to the infants in the skin‐to‐skin group (Table 3). We examined the pattern of rooting within each group as a function of time (Figure 3). Infants in the skin‐to‐skin group maintained weak lip movements from 175 min; then, their rooting increased to weak lip or tongue movements at 205 min. The pattern also changed in the mother's arms group. From 190 min, the infants showed weak lip movements followed by absence or tongue movements (Figure 3 and Table 3).

#### 3.2.4 Sucking

**FIGURE 4:**
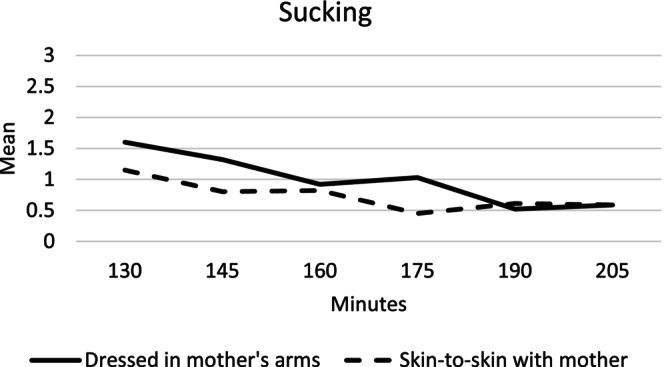
Mean sucking movements 130–205 min after birth for infants in the mother's arms and skin‐to‐skin groups.

The infants in the mother's arms group showed a higher state of sucking at 145 min and at 175 min compared to the infants in the skin‐to‐skin group (Table 3). Sucking decreased over the study period in both groups (Figure 4 and Table 3).

### 3.3 Physiological Parameters

The infants' physiological parameters: ear temperature, heart rate, and peripheral oxygen saturation were within normal range and stable and did not differ significantly between infants in the skin‐to‐skin group and the mother's arms group (Table 4).

**TABLE 4:** Infant mean temperature, heart rate, and oxygen saturation (SPO2) over the study period (130–205 min) in the mothers' arms and skin‐to‐skin groups.

Variable	Mothers' arms group	Mothers skin‐to‐skin group	*p*
	(*n* = 56)	(*n* = 39)	Mothers' arms vs. skin‐to‐skin
	Mean (SD)	Mean (SD)	
Temperature	98.3°F (0.49)	98.4°F (0.54)	0.244
	36.8°C (0.17)	36.9°C (0.17)	
Heart rate, beats/min	135.2 (13.2)	134.5 (13.6)	0.726
Oxygen saturation, %	97.5 (1.2)	97.8 (1.2)	0.644

**FIGURE 5:**
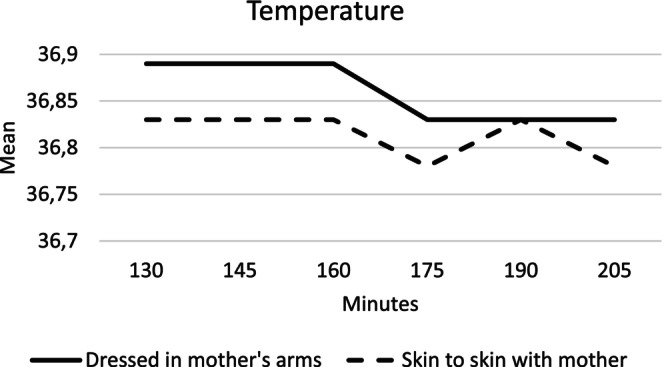
Mean temperature 130–205 min after birth for infants in the mother's arms and skin‐to‐skin groups.

During the father group allocation, the infant was randomized to be cared for in a cot, in their fathers' arms, or skin‐to‐skin with the fathers 45–120 min after birth during mother–infant separation. The infants' physiological parameters were stable with no significant differences [18] and continued to be stable in the second randomization into two groups when reunited with the mother 130–205 min after birth. Mean temperatures for infants in the skin‐to‐skin group and the mother's arms group are presented in Figure 5.

**FIGURE 6:**
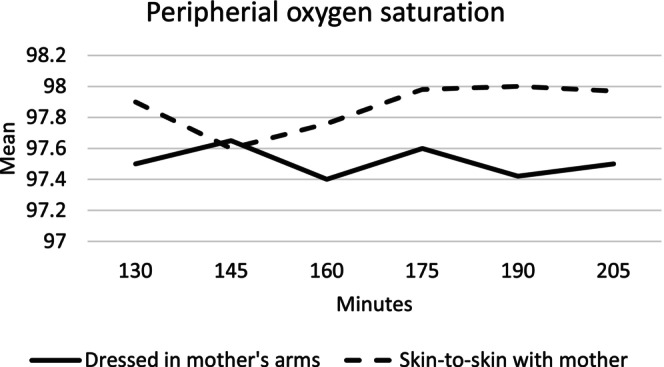
Mean peripheral oxygen saturation (%) at 130–205 min after birth for infants in the mother's arms and skin‐to‐skin groups.

The pattern of peripheral oxygen saturation for infants in the skin‐to‐skin group and the mother's arms group as a function of time is presented in Figure 6.

## 4 Discussion

The results of this study are aligned with research findings from as early as the 1970s on the importance of nonseparation of mother and child during the first hour after birth [6, 22, 23]. Our results show a significant difference in time to first breastfeeding for newborns cared for skin‐to‐skin compared to infants dressed and in the mother's arms (*p* = 0.005). During the mother–infant separation, the infants were cared for by their fathers which could have had a positive impact on the reunion with the mother and time to first breastfeeding. Newborn infants who received skin‐to‐skin contact with the father after cesarean in our previous study [19] showed some advantages over the cot and fathers' arms group when it came to establishing stable physiological parameters and wakefulness. The father, by being present and caring for the infant, may be helping to conserve the infant's energy as discussed in an RCT study from 2007 [20]. When reunited with the mother, the infant might then be more energetic and able to find the nipple more quickly for its first suckle. This can be clinically relevant and encourages parents and healthcare providers to support fathers or the other parent as primary caregivers during mother–infant separation; by doing so, they may be supporting the mother–infant reunion by facilitating the infant's prefeeding behavior during mother–infant separation [20].

In our study, the infants' wakefulness and prefeeding patterns can be compared with previous studies on infants' behavior during the first hour after birth when left alone with the mother at her chest; a high level of wakefulness continues with relaxation and awakening and ends up with sucking and then sleep [24]. The infants' physiological patterns were stable and within the normal range in the skin‐to‐skin group (*n* = 39) and in the mother's arms group (*n* = 56). This means that healthy infants, after cesarean birth can stay warm when cared for by the mother or other primary caregiver skin‐to‐skin or dressed in the mother's arms. This is in line with previous studies showing no increased risk of hypothermia when the infant is cared for by the parents and the heart rate remains stable [6, 25, 26]. Skin‐to‐skin contact has been shown in previous studies to promote cardiorespiratory adaptation after birth [27, 28]. For healthy newborn infants, initial care under a heater might not be necessary as their temperature remained stable while held [29]. The baby remaining together with the father or other caregiver during mother–infant separation is preferable [18, 19]. Qualitative findings [17] describe mothers feeling safe with the father as primary caregiver during mother–infant separation, and that the fathers wanted to be with the child. This, in turn, might support initiation of breastfeeding and bonding [17, 20]. Despite the benefits of skin‐to‐skin care, a delay or absence of skin‐to‐skin contact between mother and newborn is still reported [11, 12, 13, 14, 15, 16].

The major barriers to skin‐to‐skin contact between mother and newborn were lack of personnel, time constraints, and safety concerns [16]. Training, designated health personnel, and teamwork were identified as the key interventions likely to improve the care of the newborn after cesarean birth [16]. The focus of keeping the newborn cared for by their parents emerges from the child's right to their parents [10], the parent's desire to take care of their child [17, 18], and healthcare providers' responsibility to not separate infants from the mother, if there is no medical reason [8, 9]. When there is just cause to separate infants initially, parent and baby should be reunited as soon as possible. The baby‐friendly hospital initiative emphasizes the importance of zero separation [8, 9, 10]. With the rights of the child in mind, and the importance of parents' experiences, it is essential that healthcare professionals facilitate togetherness during this critical period [17, 18, 30, 31].

### 4.1 Strength and Limitations

The consort statement for RCT [32] was followed and the allocation of the caregiving groups has been explained clearly for transparency on the steps in the process. This adds information needed to validate the interpretation of the results. The procedures were the same for all enrolled infants, and the protocol was implemented by a trained observer conducting the naturalistic observations and performing all the measurements to ensure the reliability of the study.

The study was performed at a general public hospital in Chile from 2009 to 2012 when separation between mother and the infant was routine. The fathers and mothers therefore saw it as an opportunity to take part in this study even if the infant was not placed skin‐to‐skin with them and despite measurements being performed every 15 min [17, 18]. It is possible that the frequent measurements could have influenced the data collected and disturbed the interaction between mother and infant. The mothers included in this study were multiparas and had previous experience initiating breastfeeding. The infants were allowed to seek the breast by themselves, and the observer had extensive experience observing infants' behavior [20] and was trained to use the NBAS instrument [21]. Altogether the disturbance of the mother–infant interaction was minimized.

## 5 Conclusion

Healthy infants born by elective cesarean birth initiated breastfeeding and showed stable physiological patterns when they were cared for by their mothers 130 to 205 min after cesarean birth and when dressed in the mother's arms or skin‐to‐skin. During the mother–infant separation from birth to the reunion, the infants were cared for by their fathers. These findings support the need for healthcare providers to stop separating parents from their healthy newborn infants after a cesarean birth. It is also important to promote immediate reunion between the birthing person and the infant after a cesarean birth and to encourage parents to care for their babies as soon as possible.

## Conflicts of Interest

The authors declare no conflicts of interest.

## Data Availability

The data supporting the findings are available from the first author upon request.
